# Tnni1b-ECR183-d2, an 87 bp cardiac enhancer of zebrafish

**DOI:** 10.7717/peerj.10289

**Published:** 2020-11-04

**Authors:** Yawen Zhang, Feng Wang, Fang Wu, Youhua Wang, Xu Wang, Yonghao Gui, Qiang Li

**Affiliations:** 1 Translational Medical Center for Development and Disease, Shanghai Key Laboratory of Birth Defect, Institute of Pediatrics, Children’s Hospital of Fudan University, Shanghai, China; 2 Department of Cardiology, Children’s Hospital of Fudan University, Shanghai, China; 3 Department of Cardiology, Longhua Hospital, Shanghai University of Traditional Chinese Medicine, Shanghai, China; 4 Cancer Institute, Fudan University Shanghai Cancer Center, Shanghai, China

**Keywords:** Evolutionary conserved region, Tnni1b, Cis-regulators, Transgenic zebrafish line, Transcription factor

## Abstract

**Background:**

Several heart malformations are associated with mutations in the regulatory regions of cardiac genes. *Troponin I type 1b (tnni1b)* is important for the formation of the atrioventricular canal in zebrafish hearts; however, the regulation of *tnni1b* is poorly understand. We aimed to identify a small but functional enhancer that is distal to *tnni1b*.

**Methods:**

Evolutionary Conserved Region (ECR) Browser was used to analyze the 219 kb zebrafish and human genomes covering the *tnni1b* gene as well as the 100 kb regions upstream and downstream of *tnni1b*. Putative transcription factor binding sites (TFBSs) were analyzed using JASPAR and PROMO, and the enhancer activity was identified using zebrafish embryos and the luciferase reporter assay. A correlation analysis between the enhancer and transcription factors (TFs) was performed via TF overexpression and TFBS mutation experiments and the electrophoretic mobility shift assay (EMSA). To analyze the conservation between zebrafish and human enhancers, human DNA fragments were functionally verified. Images were captured and analyzed by fluorescence microscopy or confocal microscopy.

**Results:**

Combined with comparative analysis and functional validation, we identified a 183 bp ECR (termed tnni1b-ECR183) that was located approximately 84 kb upstream of *tnni1b* that had the heart-specific enhancer activity in zebrafish. TFBS analysis and the enhancer activity detection assay data showed that the 87 bp core region (termed tnni1b-ECR183-d2) was capable of driving specific GFP expression near the atrioventricular junction and increased luciferase expression in HEK293 and HL1 cell lines. The GFP pattern in zebrafish embryos was similar to the expression profiles of *tnni1b*. A correlation analysis showed that the enhancer activity of tnni1b-ECR183-d2 was increased when NKX2.5 (*p* = 0.0006) or JUN (*p* < 0.0001) was overexpressed and was decreased when the TFBSs of NKX2.5 (*p* < 0.0001) or JUN (*p* = 0.0018) were mutated. In addition, DNA-protein interactions were not observed between these TFs and tnni1b-ECR183-d2 in the EMSA experiment. The conservation analysis showed that tnni1b-ECR183-h179 (aligned from tnni1b-ECR183) drove GFP expression in the heart and skeletal muscles and increased the luciferase expression after NKX2.5 (*p* < 0.0001), JUN (*p* < 0.0001) or ETS1 (*p* < 0.0001) was overexpressed. Interestingly, the truncated fragment tnni1b-ECR183-h84 mainly drove GFP expression in the skeletal muscles of zebrafish and the enhancer activity decreased when NKX2.5 (*p* = 0.0028), ETS1 (*p* = 0.0001) or GATA4 (*p* < 0.0001) was overexpressed.

**Conclusions:**

An 87 bp cardiac-specific enhancer located 84 kb upstream of *tnni1b* in zebrafish was positively correlated with NKX2.5 or JUN. The zebrafish and human enhancers in this study target different tissues. The GFP expression mediated by tnni1b-ECR183-d2 is a valuable tool for marking the domain around the atrioventricular junction.

## Introduction

Congenital heart disease (CHD) is one of the most common birth defects and is associated with genetic factors, environmental factors, or both (Van der Bom et al., 2012; Fahed et al., 2013). The genetic factors, such as mutations of cardiac genes, can lead to abnormal embryonic heart development. In 2015, a large cohort study published in Science reported that either a single gene mutation or multiple gene mutations contributed to CHD pathogenesis (Homsy et al., 2015; Zhang, Liu & Tian, 2019). Notably, functional regulatory elements including promoters, enhancers and silencers, are necessary to regulate the temporal and spatial expression of target genes (Waardenberg et al., 2014; Postma, Bezzina & Christoffels, 2016), indicating that variations in regulatory regions might explain the cause of CHDs with no mutations in the coding regions. Studies of regulatory regions are mostly focused on promoter regions, which are generally located approximately 2 kb upstream of genes. Notably, several long-range regulatory elements are also functional, such as a functional enhancer that has been identified to lie within 1 Mb of the target gene *Shh* (Lettice et al., 2003). The previously reported gene-related enhancers are mostly hundreds to thousands of base pairs in length (Abbasi et al., 2010; Anwar et al., 2015). Interestingly, researchers also found that some enhancers are small, such as a 187 bp dorsal midline-specific enhancer and a 44 bp vit-2 enhancer (Charité et al., 2001; Kim, Park & Park, 2015; Goszczynski et al., 2016). These studies suggest that some distant and small enhancers are still capable of regulating gene expression.

Comparative genomics analysis is one of the most important methods to screen for functional regulatory elements. The ECR Browser (http://ecrbrowser.dcode.org) provides dynamic access to whole-genome sequences of different species, and it has been widely used to locate the regulatory elements because of its many advantages, including speed, high sensitivity and ease of operation (Ovcharenko et al., 2004; Delporte et al., 2008; Ikle, Artinger & Clouthier, 2012; Anwar et al., 2015; Sun, Chen & Peng, 2015). Moreover, the zebrafish is a valuable model organism for the identification and functional analysis of regulatory element activity (Taminato et al., 2016; Posner et al., 2017; Chan et al., 2019). Some fluorescently-labeled transgenic zebrafish lines have been widely used in clinical and scientific research (Hernández-Vega & Minguillón, 2011; Arkhipova et al., 2012; Suarez-Bregua et al., 2017). In other words, some important regulatory elements can be identified by comparative genomics analysis and functional verification in zebrafish, and one such fluorescently labeled transgenic zebrafish line will be useful for relevant researches.

Previously, researchers found that *tnni1b*^−∕−^ zebrafish embryos had several cardiac developmental abnormalities, including severe pericardial edema, heart tube deformities, endocardial ring deficiency and valve leaflet abnormalities (Shih et al., 2015; Cai et al., 2019). Data from the Gene Cards database (https://www.genecards.org/) show that the homology between zebrafish *tnni1b* and human TNNI1 is approximately 74.73(n) (Sheng & Jin, 2016), and the RNA-seq data revealed a high expression level of *tnni1b* in the zebrafish heart. Accordingly, it is essential to study the appropriate expression of *tnni1b* to enhance our understanding of the relationship between *tnni1b* and heart development. Currently, there are few studies on the regulatory network of *tnni1b*.

Therefore, in this study, a comparative analysis and functional validation were combined to investigate the functional enhancers of *tnni1b*. We successfully screened a long-range enhancer that was small but still capable of driving the specific GFP expression near the atrioventricular junction of the zebrafish heart. Considering that the enhancer in our study is small and specific, the reporter gene expression in zebrafish driven by this enhancer is a valuable tool for marking the domain around the atrioventricular junction, which means that the related transgenic zebrafish line is helpful for cardiovascular research.

## Materials and Methods

### Comparative analysis of the genomic loci of *tnni1b*

Evolutionary Conserved Region (ECR) Browser (http://ecrbrowser.dcode.org/) was used to screen for ECRs and perform genome alignment. The zebrafish (zv9) genome was set as the base genome and compared with the human (hg19) genome. To located the functional regulatory elements, a 219 kb genomic range covering the *tnni1b* gene and 100 kb regions upstream and downstream of *tnni1b* were analyzed. Furthermore, we set the ECR length parameter to be greater than 100 bp and the similarity between human and zebrafish genomes to be above 70%. ECRs with transposons and simple repeats were excluded in this study.

### Analysis of the putative TFBSs

Databases including JASPAR (http://jaspar.genereg.net/) and PROMO (http://alggen.lsi.upc.es/cgi-bin/promo_v3/promo/promoinit.cgi?dirDB=TF_8.3) were used to seek and to analyze the putative TFBSs of the enhancer sequence. As previously reported (Sandelin et al., 2004), the species was selected, and the relative profile score threshold was set to 80% to scan the target DNA sequence. Among the putative transcription factors (TFs), cardiac TFs were selected to study the correlation between them and the enhancer in our study. The regions that were covered by the most putative TFBSs were thought to be the potential core region corresponding to the original enhancer sequence.

### Construction of the TF overexpression, enhancer activity detection and mutation analysis constructs

**Figure 1:**
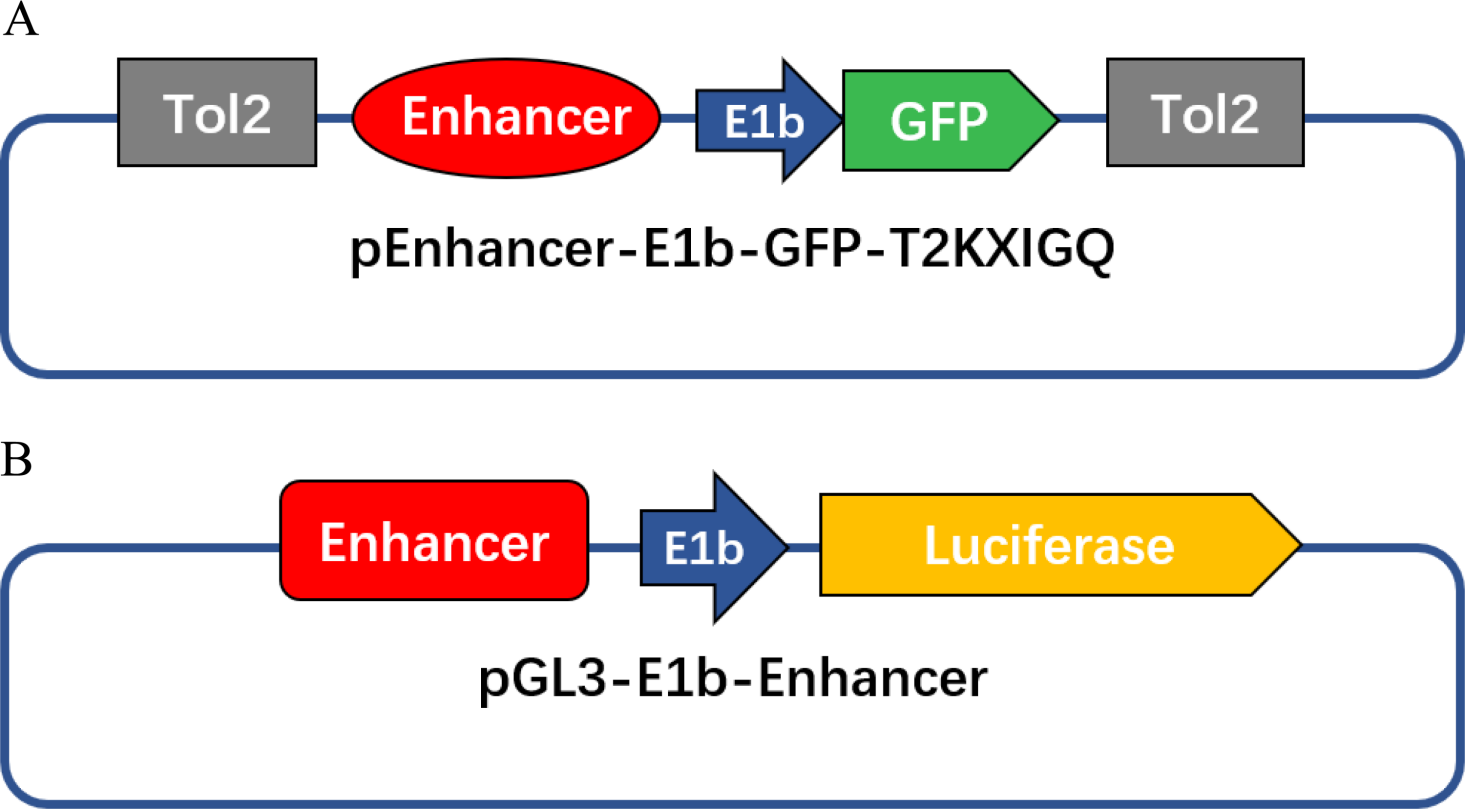
Diagrams of enhancer activity detection constructs in this study. (A) Enhancer activity detection construct used for microinjection. (B) Enhancer activity detection construct used for the luciferase reporter assay.

The ETS1 overexpression construct generated from the ETS1 cDNA sequence and the pcDNA3.1 vector was kindly provided by Professor G. Huang from the Children’s Hospital of Fudan University. The cDNA sequences of NKX2.5 and JUN, which were cloned into the pENTER vector (Vigenebio, China), were synthesized by Shanghai Sunny Biotechnology Co., Ltd. The zebrafish and human DNA sequence of the enhancer was downloaded from the ECR Browser. DNA regions of the candidate enhancers were amplified by PCR, cut with XhoI and BglII and cloned into pCNE7.04-E1b-GFP-T2KXIGQ, the enhancer activity detection vector (Li et al., 2010) (Fig. 1A). To construct a luciferase reporter plasmid (Fig. 1B), enhancer fragments were cut with KpnI and XhoI, and the original SV40 promoter of the luciferase reporter vector pGL3-promoter (Promega; USA) was replaced by E1b, which is a widely used basic promoter. We termed this new vector pGL3-E1b. In the mutated enhancer constructs, a single base in the TFBSs was mutated but was not introduced a new heart-related TFBS. The primer sequences used for PCR amplification of the enhancer activity detection and mutation analysis constructs are listed in Table 1.

**Table 1:** Primer sequences used for PCR amplification of the enhancer activity detection and TFBS mutation analysis constructs.

**Constructs**	**Sequences (5′–3′)**
ptnni1b-ECR183-E1b- GFP-T2KXIGQ	F: agagctcgagctgacagatagctgctgccggtagag R: agagagatcttgtctctttccctctgcagttgtctg
ptnni1b-ECR183-d1-E1b- GFP-T2KXIGQ	F: tcgagccaggagagaggagacgagcggttggagaggctggagatgctgcgagcattggtttcatcgctgtcataa R: gatcttatgacagcgatgaaaccaatgctcgcagcatctccagcctctccaaccgctc gtctcctctctcctggc
ptnni1b-ECR183-d2-E1b- GFP-T2KXIGQ	F: tcgagctgacagatagctgctgccggtagaggaaggagcatctccggcttggagacgaggactggactgaccgtggcgccaggagagaggaga R: gatctctcctctctcctggcgccacggtcagtccagtcctcgtctccaagccggagat gctccttcctctaccggcagcagctatctgtcagc
ptnni1b-ECR183-d3-E1b- GFP-T2KXIGQ	F: tcgagtgacagatagctgctgccggtagaggaaggagcatctccgga R: gatctccggagatgctccttcctctaccggcagcagctatctgtcac
ptnni1b-ECR183-h179-E1b- GFP-T2KXIGQ	F: agagctcgagccgagcggcagctcccacccacaga R: agagagatcttgccttctgctctccacccagctccc
ptnni1b-ECR183-h84-E1b- GFP-T2KXIGQ	F: tcgagccgagcggcagctcccacccacagagggcgcgtcaccagcctgcagccgcggactggactggccatagcgccatgacagagggga R: gatctcccctctgtcatggcgctatggccagtccagtccgcggctgcaggctggtgacgcgccctctgtgggtgggagctgccgctcggc
pGL3-E1b-tnni1b- ECR183	F: agagggtaccctgacagatagctgctgccggtagag R: agagctcgagtgtctctttccctctgcagttgtctg
pGL3-E1b-tnni1b- ECR183-d2	F: cctgacagatagctgctgccggtagaggaaggagcatctccggcttggagacgaggactggactgaccgtggcgccaggagagaggagc R: tcgagctcctctctcctggcgccacggtcagtccagtcctcgtctccaagccggagat gctccttcctctaccggcagcagctatctgtcagggtac
pGL3-E1b-tnni1b- ECR183-h179	F: agagggtaccccgagcggcagctcccacccacaga R: agagctcgagtgccttctgctctccacccagctccc
pGL3-E1b-tnni1b- ECR183-h84	F: cccgagcggcagctcccacccacagagggcgcgtcaccagcctgcagccgcggactggactggccatagcgccatgacagaggggc R: tcgagcccctctgtcatggcgctatggccagtccagtccgcggctgcaggctggtga cgcgccctctgtgggtgggagctgccgctcggggtac
pGL3-E1b-tnni1b- ECR183-d2-NKX2.5-mut	F: cctgacagatagctgctgccggtagaggaaggagcatc**g**(t)ccggcttggagacgaggactggactgaccgtggcgccaggagagaggagc R: tcgagctcctctctcctggcgccacggtcagtccagtcctcgtctccaagccgg**c**(a) gatgctccttcctctaccggcagcagctatctgtcagggtac
pGL3-E1b-tnni1b- ECR183-d2-JUN-mut	F: cctga**t**(c)agatagctgctgccggtagaggaaggagcatctccggcttggagacgaggactggac**g**(t)gaccgtggcgccaggagagaggagc R: tcgagctcctctctcctggcgccacggtc**c**(a)gtccagtcctcgtctccaagccgga gatgctccttcctctaccggcagcagctatct**a**(g)tcagggtac

**Notes.**

F:Forward primer sequence.

R:Reverse primer sequence.

Mutant bases are shown as bolded text, original bases are in parentheses.

### Zebrafish maintenance, microinjection, and transgenic line generation

The animal protocols were approved by the Institutional Animal Care and Use Committee of Children’s Hospital of Fudan University (approval reference number: EK201873). Zebrafish strains used in this study included TU wild-type and transgenic line *Tg* (myl7: mCherry) (kindly provided by Professor Q. Jiang at Zhongshan Hospital of Fudan University), which was a transgenic line with cardiomyocyte-specific red fluorescence driven by the promoter of *myl7*. Embryos for fluorescence observation were incubated in blue egg water containing 0.003% phenylthiourea to inhibit pigmentation. In brief, a microinjection solution containing 25∼50 ng/µl DNA from the candidate regulatory region, 25 ng/µl tol2 mRNA and 0.1% phenol red was injected into fertilized zebrafish embryos at the single cell stage. Approximately 50 embryos on average survived at 24 h post-fertilization (hpf). F0 fish with positive green fluorescent expression were outcrossed with TU wild-type fish to obtain the F1 generation, and the F2 generation was back-crossed offspring of F1. For the co-localization analysis, *Tg* (myl7: mCherry), which was labeled by red fluorescence specifically in cardiomyocytes, was crossed with the transgenic zebrafish line in this study.

### Cell culture, transfection, and luciferase reporter assay

The HEK293 and HL1 cell lines were purchased from Shanghai Fuheng Biotechnology Co., Ltd. Cells were maintained in 60 mm tissue culture grade dishes at 37 °C in 5% CO_2_. The culture medium was DMEM, high glucose, pyruvate (Cat. No. 11995065; Invitrogen; USA) supplemented with 10% fetal bovine serum (Cat. No. 10099141; Invitrogen; USA), 100 U/ml penicillin (Invitrogen; USA) and 100 mg/ml streptomycin (Invitrogen; USA). HEK293 and HL1 cells were plated in 96-well plates at 5 ×10^3^ cells/well and 8 ×10^3^ cells/well, respectively. For the enhancer activity detection assay, both HEK293 and HL1 cells were transfected with a 100 ng/well enhancer activity detection construct using Lipofectamine 3000 reagent (Cat. No. L3000015; Invitrogen; USA) according to the manufacturer’s instructions after 24 h of culture. For the TF overexpression experiment, both the enhancer activity detection construct (100 ng/well) and TF overexpression construct (100 ng/well) were cotransfected into HEK293 cells, while for the TFBS mutation experiment, only the enhancer mutation analysis construct (100 ng/well) was transfected. After 48 h of transfection, cells were collected to obtain the lysate, and the firefly and Renilla luciferases were detected by a Dual-luciferase Reporter Assay System (Cat. No. E1910; Promega; USA). The ratio of the firefly luciferase value and the *Renilla* luciferase value was the relative luciferase activity. Experiments were repeated for three times. For the EMSA experiment, HEK293 cells were maintained in 60 mm dishes. Cells were transfected when they reached 70% confluence. In total, 10 µg TF overexpression construct was transfected into the cells, the cells were incubated for 48–72 h after transfection, and protein extraction was performed.

### Protein extraction and electrophoretic mobility shift assay (EMSA)

A Nuclear and Cytoplasmic Protein Extraction Kit (Beyotime; Shanghai) and quantified proteins from a BCA Protein Assay Kit (Beyotime; Shanghai) were used for extraction and concentration determination. Protocols followed the manufacturer’s instructions. The procedure for EMSA was described in the instructions for a LightShift^®^ Chemiluminescent EMSA Kit (Thermo Scientific; USA). In brief, a control Epstein-Barr nuclear antigen (EBNA) system included three reactions: specific binding reactions, competition reactions and negative reactions that contained no protein extract, while the test system included additional TFBS mutation reactions. The biotin end-labeled target DNA, unlabeled DNA and TFBS mutation DNA were amplified by PCR. The unlabeled DNA sequences were the same as the biotin-labeled sequences, and primers of the oligos for biotin-labeled target DNA and TFBS mutation DNA are listed in Table S1. Next, 0.1 µM biotin-labeled target DNA (2 µl), 10x binding buffer (2 µl), 1 µg/µl poly (dI-dC) (1 µl), 50% glycerol (1 µl), 1% NP-40 (1 µl) and 100 mM MgCl2 (1 µl) were in each reaction, and 10 µM unlabeled DNA (4 µl) was in a competition reaction while 10 µM TFBS mutated DNA (4 µl) was in a TFBS mutation reaction. All reactions contained 10 µg of protein extract except the negative control. After incubation at room temperature for 30 min, 20 µl of each binding sample was loaded onto a 6% polyacrylamide gel. A 0.45 µm nylon membrane was used for electrophoretic transfer on ice with 0.5X TBE at 380 mA (∼100 V) for 30 min. Biotin-labeled DNA was detected by chemiluminescence after the membrane was blocked for 15 min and washed 5 times (5 min/wash).

### Fluorescent image processing and analysis

The expression of GFP in microinjected zebrafish embryos was detected by a fluorescence microscope (Leica 205C; Germany) at 24 hpf, 48 hpf and 72 hpf, and expression in the injected group was compared with that in the noninjected group, while impurities on the microscope lens shown as black dots. Positive embryos with the specific expression of GFP in heart tissue were maintained for a follow-up study. The different time points of observation of the transgenic zebrafish lines included 24 hpf, 48 hpf, 72 hpf, 96 hpf, 5 days postfertilization (dpf) and 10 dpf. The distribution of the fluorescent expression in the embryos of the stable transgenic zebrafish line crossed with *Tg* (myl7: mCherry) was observed by confocal microscopy (Leica TCS SP8 X; Germany). All images were adjusted by the professional software ImageJ and Adobe Illustrator CS6.

### Statistical analysis

Mean values and standard errors were calculated using standard methods. All experiments were repeated at least 3 times. GraphPad Prism 6.0 was used for all statistical analyses. Student’s t test was used for the statistical analysis between two groups. When comparing multiple experiments, a One-way ANOVA was used to test for homogeneity of variance. Bonferroni’s test was used to correct for multiple comparisons based on confidence intervals and significance. A value of *p* < 0.05 was considered to be statistically significant.

## Results

### Identification of a 183 bp ECR located 84 kb upstream of *tnni1b*

**Figure 2:**
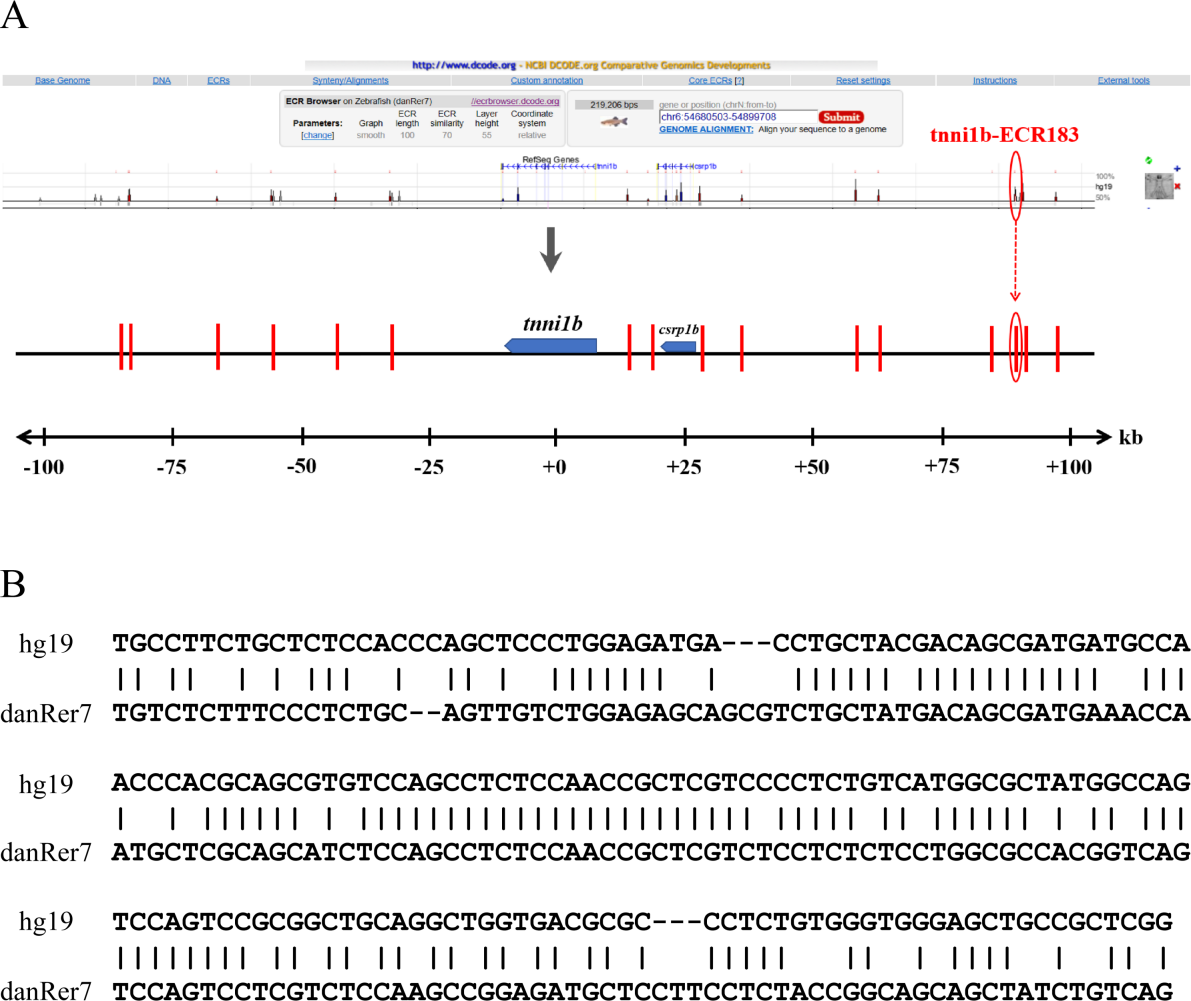
Comparative analysis of the genomic loci of *tnni1b*. (A) ECR Browser and parameter set between zebrafish and human genomes around the 219 kb genome range encompassing the *tnni1b* gene and 100 kb regions upstream and downstream of *tnni1b*. Genes are shown in blue, and the 16 ECRs are shown in the red segment. (B) Overlapping alignment block: sequences of hg19-chr1:201687497-201687890 and zv9-chr6:54883657-54884033.

**Figure 3:**
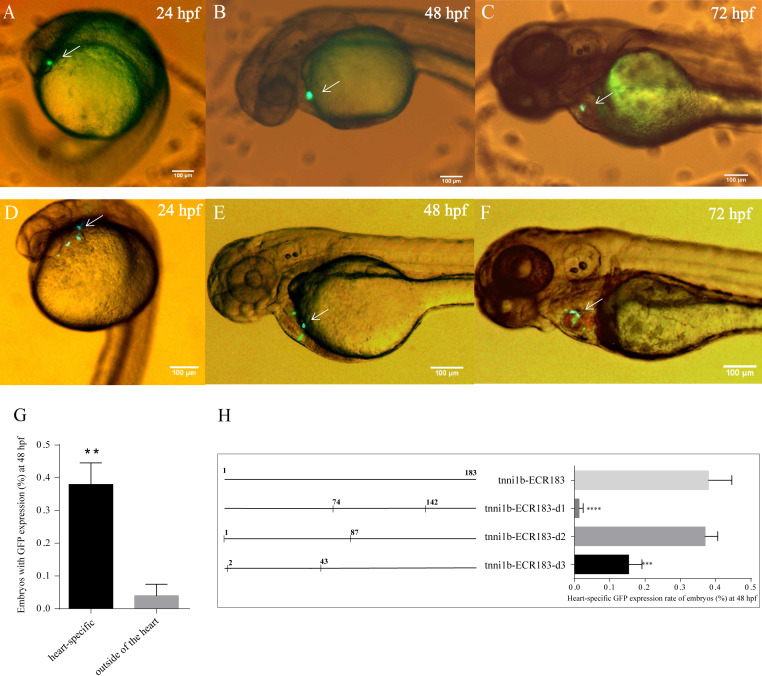
GFP expression after the injection of zebrafish enhancers. Lateral views of zebrafish embryos with heart-specific GFP expression after injection with tol2 mRNA and tnni1b-ECR183 (A–C) or tnni1b-ECR183-d2 (D–F), scale bars = 100 µm. The average ratios of heart-specific GFP expression embryos to the total surviving embryos were 10/45 (22%) at 24 hpf, 15/41 (37%) at 48 hpf, and 15/39 (38%) at 72 hpf in the tnni1b-ECR183 group and 7/59 (12%) at 24 hpf, 18/48 (38%) at 48 hpf and 16/43 (37%) at 72 hpf in the tnni1b-ECR183-d2 group. (G) Rate of embryos with GFP expression in different tissues at 48 hpf after injection with tnni1b-ECR183. (H) Rate of embryos with heart-specific GFP expression from transient injections of tnni1b-ECR183 and each truncated enhancer with tol2 transposase at 48 hpf. A *t*-test was used for statistical analyses between the two groups (G). One-way ANOVA was used to test for homogeneity of variance, and Bonferroni’s test was used to correct for multiple comparisons (H). ** *p* < 0.01; *** *p* < 0.001; **** *p* < 0.0001, *n* = 3.

To investigate the functional enhancers of *tnni1b*, we performed a comparative analysis on the 219 kb zebrafish genomic region encompassing the *tnni1b* gene and the 100 kb regions upstream and downstream of *tnni1b.* In total, 16 ECRs were in the target genomic region (Fig. 2A). Information on these ECRs is listed in Table S2. According to the screening criteria, we found that one 183 bp ECR (location: zv9-chr6: 54883657-54883839, percent identity: 71.6%) was located at approximately 84 kb upstream of *tnni1b* (Fig. 2A). An overlapping alignment block is shown in Fig. 2B. After the injection of the enhancer DNA construct and tol2 mRNA, the GFP expression driven by this 183 bp zebrafish DNA fragment was mainly detected in the heart (Figs. 3A–3C), and fewer and weaker GFP signals were detected outside of the heart, such as in the head or back (Fig. S1). The average ratios of heart-specific GFP expression embryos to total surviving embryos were 10/45 (22%) at 24 hpf, 15/41 (37%) at 48 hpf, and 15/39 (38%) at 72 hpf. The rate of embryos with GFP expression in the heart and other tissues at 48 hpf is shown in Fig. 3G. Notably, among the total embryos with GFP expression, more than 70% showed heart-specific fluorescent expression at different time points. We termed this ECR tnni1b-ECR183.

### The core region, tnni1b-ECR183-d2, is only 87 bp

**Figure 4:**
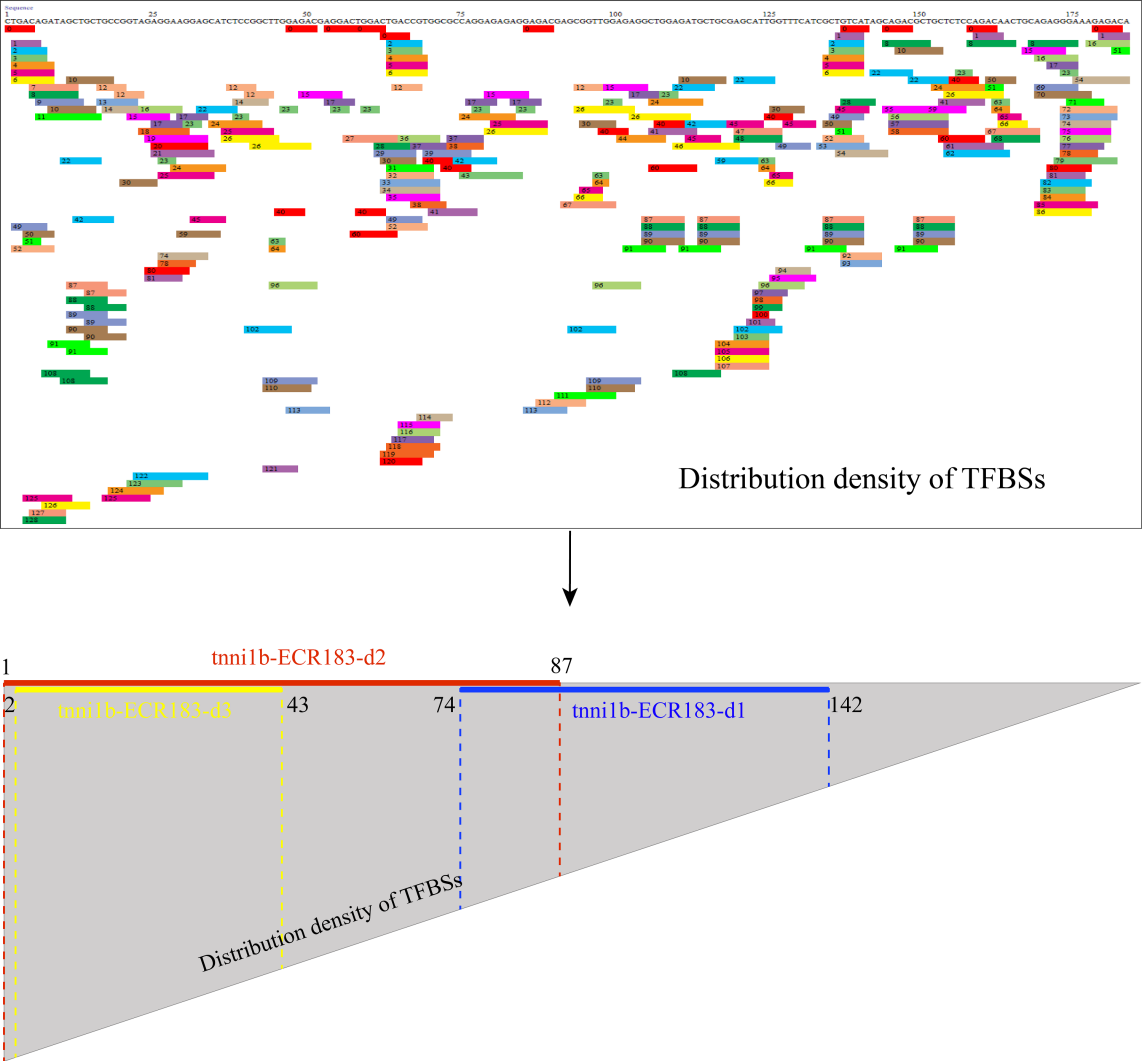
Putative TFBS analysis of tnni1b-ECR183 by PROMO and the specific positions of truncated enhancer fragments including tnni1b-ECR183-d1, tnni1b-ECR183-d2, and tnni1b-ECR183-d3. TFBSs are displayed in fragments of different colors and numbers. The area division of the triangle represents the distribution density of TFBSs.

We performed deletion analysis to find the core region of tnni1b-ECR183 based on the TF binding affinity analysis described previously (Hallikas et al., 2006). As shown in Fig. 4, the results of PROMO analysis showed that approximately 112 putative binding conditions and TFs mostly bound to the nucleotide sequence 1 to 142, which was the potential core region of this 183 bp sequence. Combined with the 388 TFBSs sought by JASPAR, we found that 60% of TFBSs covered the range in the nucleotide sequence from 1 to 87, 26% from the nucleotide sequence 74 to 142 and only 20% from the nucleotide sequence 142 to 183. We then focused on the analysis of the nucleotide sequence 1 to 87 and found that the putative TFs mainly bound to the nucleotide sequence 2 to 43. We therefore constructed three enhancer activity detection constructs that contained truncated enhancer DNA fragments tnni1b-ECR183-d1 (nucleotide sequence 74 to 142), tnni1b-ECR183-d2 (nucleotide sequence 1 to 87) and tnni1b-ECR183-d3 (nucleotide sequence 2 to 43) (Fig. 4). After comparing the rate of embryos with heart-specific GFP expression between the tnni1b-ECR183 and each truncated enhancer group at 48 hpf (Fig. 3H), we found that tnni1b-ECR183-d2 was also capable of driving the heart-specific expression of GFP (*p* > 0.9999). Images of embryos injected with tnni1b-ECR183-d2 at 24 hpf, 48 hpf and 72 hpf are shown in Figs. 3D–3F, and the corresponding videos are respectively shown in Videos S1, S2 and S3. Moreover, the average ratios of heart-specific GFP expression in total live embryos were 7/59 (12%) at 24 hpf, 18/48 (38%) at 48 hpf and 16/43 (37%) at 72 hpf. Similar to tnni1b-ECR183, among the total embryos with GFP expression, the percentage of embryos with heart-specific GFP expression driven by tnni1b-ECR183-d2 was over 70%. In addition, tnni1b-ECR183-d3 also drove GFP expression in some zebrafish embryos, but the fluorescence intensity was weaker and the ratio of heart-specific GFP expression was less than that of tnni1b-ECR183 (*p* = 0.0005) (Fig. 3H). These results suggest that the core region of tnni1b-ECR183 is tnni1b-ECR183-d2, which is 87 bp in size, and it also has the enhancer activity to drive the specific expression of GFP in the zebrafish heart.

### Tnni1b-ECR183-d2 drives the specific expression of GFP near the atrioventricular junction

**Figure 5:**
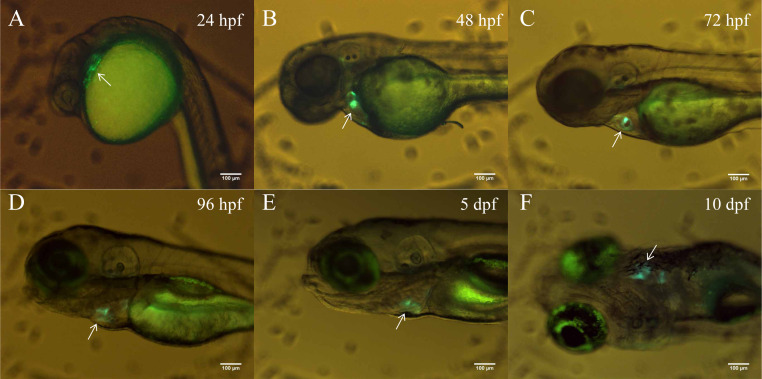
The heart-specific GFP expression in the stable transgenic zebrafish line *Tg* (tnni1b-ECR183-d2: GFP). (A–E) Lateral views of zebrafish embryos with heart-specific GFP expression at 24 hpf, 48 hpf, 72 hpf, 96 hpf and 5 dpf. (F) Ventral view of zebrafish embryo with heart-specific GFP expression at 10 dpf. Scale bars = 100 µm.

**Figure 6:**
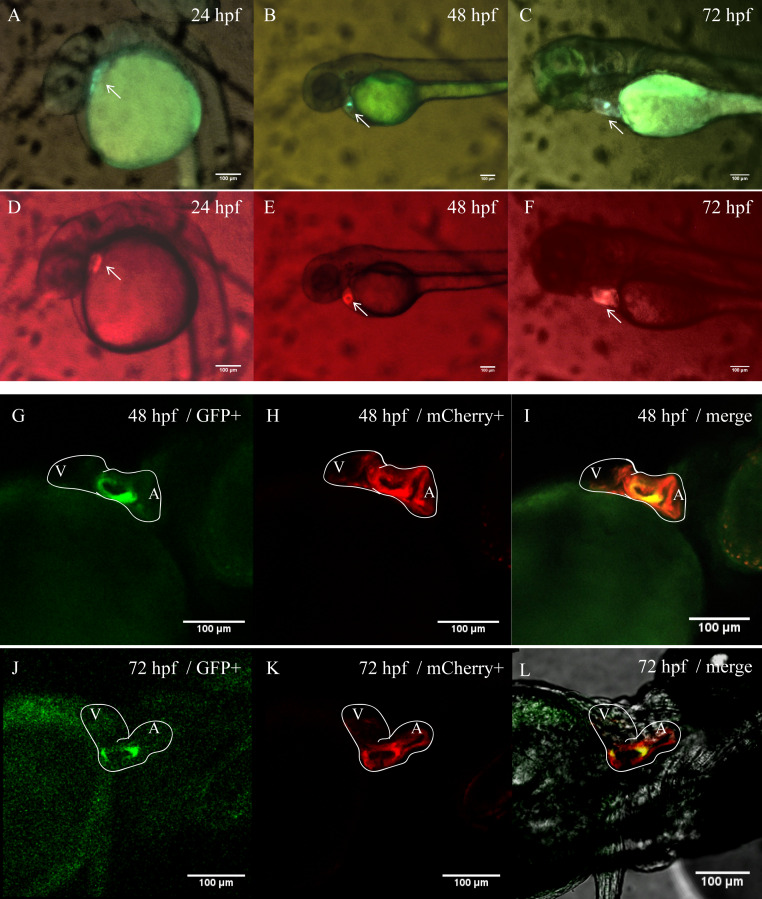
Heart-specific GFP expression in zebrafish embryos of *Tg* (tnni1b-ECR183-d2: GFP; myl7: mCherry). (A–F) Lateral views of zebrafish embryos with GFP expression driven by tnni1b-ECR183-d2 (A–C) and those with mCherry expression driven by the promoter of *myl7* (D–F). (G–L) Confocal images of ventral views of these embryos with GFP expressed in green (G, J), mCherry expressed in red (H, K) and the overlapping part expressed in yellow (I, L). White line, heart outline; A, atrium; V, ventricle. Scale bars = 100 µm.

In order to better clarify the spatial and temporal distribution of heart-specific fluorescent expression driven by tnni1b-ECR183-d2, we generated the transgenic line *Tg* (tnni1b-ECR183-d2: GFP) and observed the expression of GFP in offspring at different time points. We found that the heart-specific green fluorescence was detectable at 24 hpf, 48 hpf, 72 hpf, 96 hpf, 5 dpf and 10 dpf (Fig. 5). *Myl7* is a typical cardiac gene, and the fluorescence reporter gene that is driven by the promoter of *myl7* is often used to mark cardiomyocytes (Shi et al., 2017; Elworthy et al., 2019; Fricke et al., 2020). To better observe the specific position of fluorescent expression driven by tnni1b-ECR183-d2 in the heart, we then crossed *Tg* (tnni1b-ECR183-d2: GFP) with *Tg* (myl7: mCherry) to generate the transgenic line *Tg* (tnni1b-ECR183-d2: GFP; myl7: mCherry), and perform co-localization analysis by comparing the difference between the green and red fluorescence expressions. As shown in Figs. 6A–6F, both the green fluorescence driven by tnni1b-ECR183-d2 and the red fluorescence driven by the promoter of cardiac gene *myl7* were detected in the heart, indicating that tnni1b-ECR183-d2 is a cardiac enhancer. Zebrafish heart began to loop at 30 hpf, exhibited an obvious ‘s’-shape until 36 hpf, and gradually expanded to the recognizable atrium and ventricle chambers around 48 hpf (Kimmel et al., 1995; Grant et al., 2017). We used a confocal microscope to capture the heart images of zebrafish embryos at 48 hpf and 72 hpf to facilitate further comparisons and analyses. As shown in Figs. 6G–6L, in zebrafish embryos of *Tg* (tnni1b-ECR183-d2: GFP; myl7: mCherry), GFP driven by tnni1b-ECR183-d2 is expressed in green fluorescence and mCherry driven by the promoter of cardiomyocyte-specific gene *myl7* is expressed in red fluorescence. The overlapping yellow part represents the cardiomyocytes expressing both GFP and mCherry, which are mostly in the atrium and ventricle boundary. Taken together, these results show that tnni1b-ECR183-d2 is a heart-specific enhancer, and the GFP expression driven by this enhancer is around the atrioventricular junction.

### Analysis of the enhancer activity of tnni1b-ECR183-d2 by a luciferase assay

**Figure 7:**
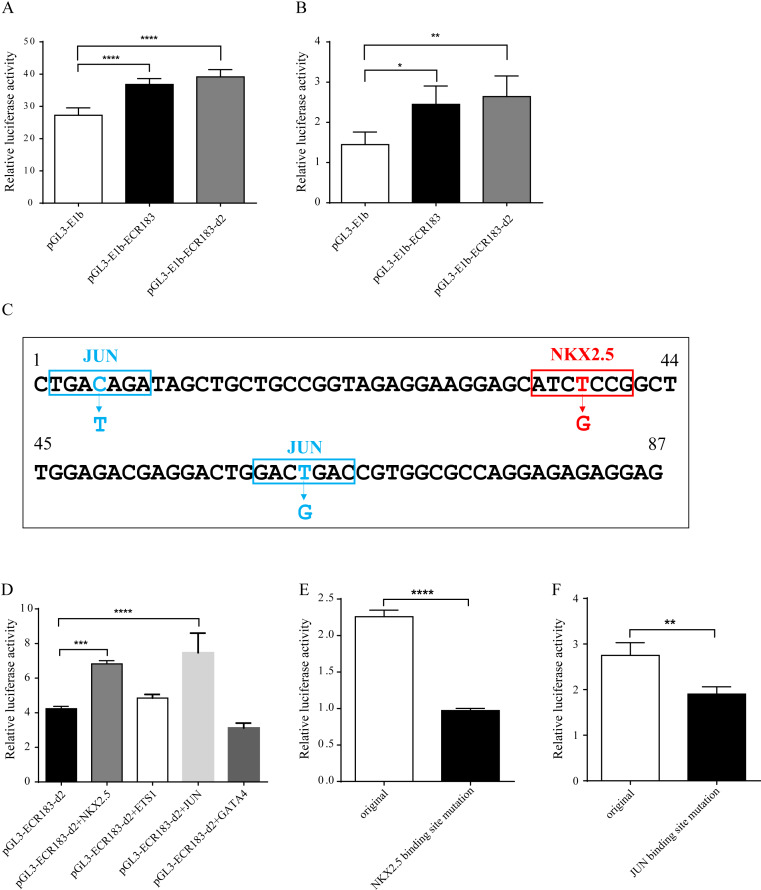
Identification of the enhancer activity and analysis of putative TFBSs. Enhancer activity identification of tnni1b-ECR183 and tnni1b-ECR183-d2 by the luciferase assay in the HEK293 (A) and HL1 cell lines (B). (C) Putative TFBS positions and mutations. Analysis of the enhancer activity of tnni1b-ECR183-d2 after putative TFs were overexpressed (D) or after NKX2.5 (E) and JUN (F) binding sites were mutated. One-way ANOVA was used to test for homogeneity of variance, and Bonferroni’s test was used to correct for multiple comparisons (A, B, D). A *t*-test was performed for statistical analyses between two groups (E, F). * *p* < 0.05; ** *p* < 0.01; *** *p* < 0.001; **** *p* < 0.0001, *n* = 3.

We also performed functional analysis of tnni1b-ECR183 and tnni1b-ECR183-d2 in vitro. We generated the enhancer activity detection constructs and transfected them into HEK293 cells and HL1 cells. As shown in Figs. 7A–7B, higher luciferase activity was detected in the tnni1b-ECR183 group (in HEK293 cells: *p* < 0.0001; in HL1 cells: *p* = 0.0199) and tnni1b-ECR183-d2 group (in HEK293 cells: *p* < 0.0001; in HL1 cells: *p* = 0.0075) than in the pGL3-E1b group. Combined with the GFP expression in the zebrafish heart driven by tnni1b-ECR183-d2, these results of in vivo and in vitro verification showed that this 87 bp ECR was a functional heart-specific enhancer.

### Tnni1b-ECR183-d2 has positive correlations with NKX2.5 or JUN

Among the putative TFs, four cardiac TFs including NKX2.5, GATA4, ETS1 and JUN were closely related to heart development according to previously published studies. After performing the TF overexpression analysis, we found that the enhancer activity of tnni1b-ECR183-d2 increased significantly when NKX2.5 (*p* = 0.0006) or JUN (*p* < 0.0001) was overexpressed (Fig. 7D). According to the website tools and mutation principles introduced in the Methods section, we then performed further experiments on TFBS mutations (Fig. 7C). The enhancer activity was significantly decreased when the putative binding sequence of NKX2.5 was mutated from ATCTCCG to ATCGCCG (*p* < 0.0001) (Fig. 7E); those of JUN were mutated from TGACAGA and GACTGAC to TGATAGA and GACGGAC, respectively (*p* = 0.0018) (Fig. 7F). In addition, no DNA-protein interactions between these TFs and tnni1b-ECR183-d2 were detected by autoradiography in the EMSA experiment (Fig. S2). These results suggest that TFs, including NKX2.5 and JUN, might have a positive indirect effect on the enhancer function of tnni1b-ECR183-d2.

### Functional analysis of human enhancers

**Figure 8:**
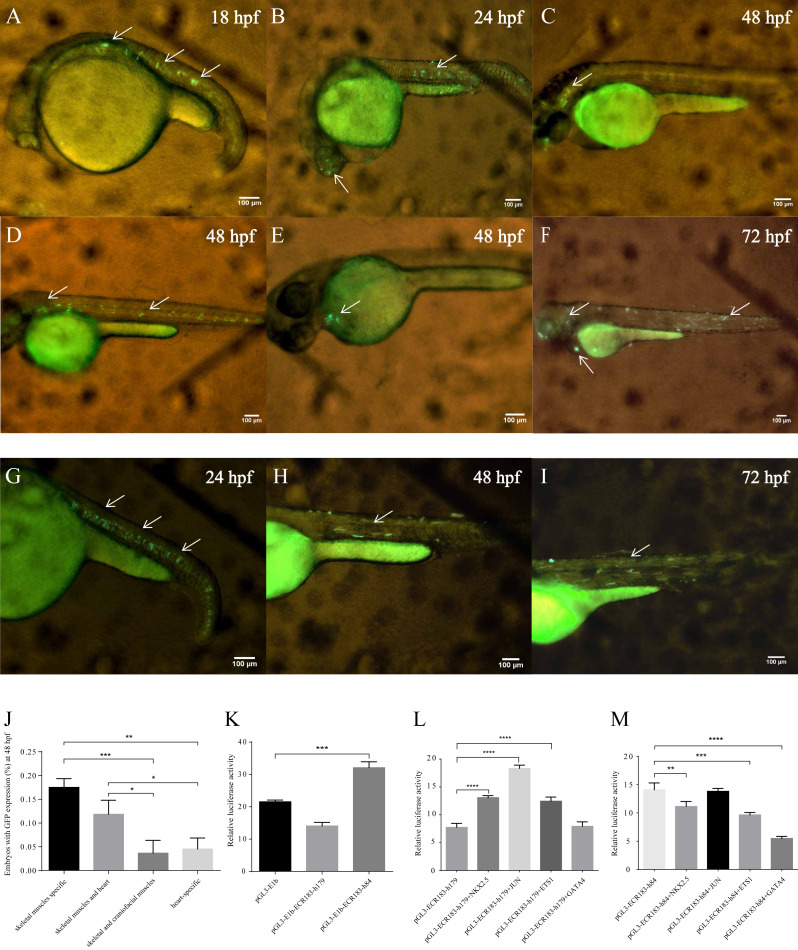
Functional analysis of human enhancers. (A–F) Lateral views of zebrafish embryos with GFP expression after injection with tol2 mRNA and tnni1b-ECR183-h179. (G–I) Lateral views of zebrafish embryos with GFP expression after injection with tol2 mRNA and tnni1b-ECR183-h84. The arrowheads indicate GFP expression in the skeletal muscles (A, B, D, F, G, H, I), craniofacial muscles (B, C, D, F) and hearts (E, F), scale bars = 100 µm. Rate of embryos with GFP expression in different tissues after injection with tnni1b-ECR183-h179 (J). Enhancer activity identification of tnni1b-ECR183-h179 and tnni1b-ECR183-h84 in HEK293 cell lines (K). Analysis of the enhancer activity of tnni1b-ECR183-h179 (L) and tnni1b-ECR183-h84 (M) after putative TFs were overexpressed. One-way ANOVA was used to test for homogeneity of variance, and Bonferroni’s test was used to correct for multiple comparisons. * *p* < 0.05; ** *p* < 0.01; *** *p* < 0.001; **** *p* < 0.0001, *n* = 3.

To analyze the conservation between zebrafish and human enhancers, we aligned tnni1b-ECR183 and tnni1b-ECR183-d2 to human DNA sequences located 288.6 kb upstream of TNNI1, which were termed tnni1b-ECR183-h179 (location: hg19-chr1: 201687497-201687675) and tnni1b-ECR183-h84 (location: hg19-chr1: 201687592-201687675), respectively. After functionally verifying these human enhancer regions, we found that zebrafish embryos injected with tol2 mRNA and tnni1b-ECR183-h179 showed GFP expression in skeletal muscles alone or in the heart, skeletal and craniofacial muscles (Figs. 8A–8F). The rates of embryos with GFP expression in different tissues suggested that tnni1b-ECR183-h179 dominantly drove GFP expression in the skeletal muscles, the heart, or both (Fig. 8J). Interestingly, those embryos injected with tnni1b-ECR183-h84 showed GFP expression mainly in the skeletal muscles (Figs. 8G–8I). As shown in Fig. 8K, luciferase expression was increased by tnni1b-ECR183-h84 (*p* = 0.0001), while expression driven by tnni1b-ECR183-h179 did not show a significant increase. To determine whether the same TFBSs tested in the zebrafish enhancer regions were conserved, we further analyzed the enhancer activity of tnni1b-ECR183-h179 and tnni1b-ECR183-h84 after putative TFs were overexpressed. Figure 8L shows that the luciferase expression level was significantly increased by tnni1b-ECR183-h179 when NKX2.5 (*p* < 0.0001), JUN (*p* < 0.0001) or ETS1 (*p* < 0.0001) was overexpressed. However, the enhancer activity of tnni1b-ECR183-84 decreased when NKX2.5 (*p* = 0.0028), ETS1 (*p* = 0.0001) or GATA4 (*p* < 0.0001) was overexpressed (Fig. 8M), which suggested a weak correlation between this enhancer and the heart. The above results indicate that the human enhancer tnni1b-ECR183-h179 is related to the heart and skeletal muscles while tnni1b-ECR183-h84 is a skeletal muscle-specific enhancer.

## Discussion

Currently, etiology analyses of CHDs focus mostly on the variants in the coding regions of several typical heart-related genes (McCulley & Black, 2012). Interestingly though, the findings of published studies that used targeted gene panels or exome sequencing implicate that approximately 90% of sporadic CHD patients do not carry mutations in exon regions (Blue et al., 2017), indicating that other causes including unknown pathogenic genes and variations in regulatory regions, should not be ignored. Indeed, mutations in the regulatory regions of cardiac genes can also cause several heart defects (Pang et al., 2012; Smemo et al., 2012; Wu et al., 2012; Gu et al., 2017), such as the mutation rs118026695 within the promoter of NKX2.5 and the homozygous mutation in an enhancer, which is located approximately 90 kb downstream of TBX5. In this study, we found that an enhancer located 84 kb upstream of *tnni1b* was capable of mediating specific GFP expression in zebrafish hearts. Notably, this heart-specific enhancer in this study was only 87 bp in size, and the enhancer in our previous study was also small (42 bp) (Wang et al., 2019). Taken together, we think that small enhancers that are remote from cardiac genes are still functional; thus, future studies are needed to explore the specific function of these enhancers on target genes and their roles in the development or pathophysiology of the heart.

Fluorescently-labeled transgenic zebrafish lines, which are mediated by tissue-specific regulatory elements, are useful in pathophysiology studies (Da Silva Lopes et al., 2011; Shi et al., 2017). For instance, the dynamics of centrosomes in radial glia neural progenitors were observed by performing time-lapse imaging on the zebrafish brain, where the nucleus was labeled by GFP and the plasma membrane was labeled by tdTomato (Yu et al., 2016). In this study, the transgenic zebrafish line *Tg* (tnni1b-ECR183-d2: GFP) was used to track the heart-specific fluorescent expression in real-time during zebrafish development. This result suggests that the GFP expression mediated by tnni1b-ECR183-d2 can be used as a heart-specific marker, and such a transgenic zebrafish line is expected to be useful for cardiovascular research.

To identify the TFs that interacted with tnni1b-ECR183-d2, we performed TFBS analysis using JASPAR and PROMO and selected four TFs associated with heart development. Combined with the TF overexpression and TFBS mutation experimental results, we found that NKX2.5 or JUN was necessary for enhancer activation of tnni1b-ECR183-d2. However, the EMSA results did not show direct interactions between them. One of the explanations of this result is that the specific binding reactions between TFs and enhancers are not strong enough to be detected. Moreover, given that the gene regulatory network is complex and that TFs could collaborate with each other (Csumita et al., 2020; Theeuwes et al., 2020), indirect interactions might exist between the above TFs and enhancers in our study.

In addition, we compared the heart-specific fluorescent expression in our study with the gene expression pattern of in situ hybridization on ZFIN (http://zfin.org/) to investigate the target genes that might be regulated by tnni1b-ECR183-d2. Researchers have found that *tnni1b* is expressed in the heart rudiment and heart tube of zebrafish at 24 hpf and 48 hpf respectively, and becomes stable in the heart after 60 hpf (http://zfin.org/ZDB-GENE-041212-37/expression). This expression pattern is consistent with the spatiotemporal expression of green fluorescence driven by tnni1b-ECR183-d2. In addition, it was reported that *tnni1b* ^−∕−^ zebrafish showed developmental defects of the endocardial ring at the atrioventricular junction (Cai et al., 2019), and the confocal images in our study showed the strong expression of green fluorescence near the atrioventricular junction. This evidence indicates that *tnni1b* might be the target gene regulated by tnni1b-ECR183-d2.

To analyze the conservation between zebrafish and human enhancers, we functionally verified the human enhancers tnni1b-ECR183-h179 and tnni1b-ECR183-h84, which were aligned from tnni1b-ECR183 and tnni1b-ECR183-d2. In the human genome, TNNI1 encodes the slow skeletal muscle isoform of TnI (ssTnI), while TNNI3 encodes the cardiac isoform of TnI (cTnI). Previous studies have demonstrated that the TnI isoforms are functionally conservative and ssTnI is expressed in both skeletal muscles and hearts in fetuses (Saggin et al., 1989; Sasse et al., 1993). Similarly, in this study, the GFP expression in zebrafish embryos observed after injection showed that tnni1b-ECR183-h179, which is located at 288.6 kb upstream of TNNI1, is related to the heart and skeletal muscles. However, the luciferase assay did not identify obvious enhancer activity of tnni1b-ECR183-h179. Considering the complex gene regulatory network and the collaboration between TFs (Csumita et al., 2020), tnni1b-ECR183-h179 might bind to repressive TFs to decrease the expression of luciferase in this study. The truncated fragment tnni1b-ECR183-h84 showed skeletal muscle-specific enhancer activity. Combined with the findings that the zebrafish DNA fragments in this study were heart-specific enhancers, we hypothesize that the zebrafish and human enhancers in this study target different tissues.

Here, a small (87 bp) heart-specific enhancer of zebrafish that lies 84 kb upstream of *tnni1b* was identified. In the future, the spatiotemporal activity of the new enhancer reported in this study should be thoroughly studied in mice or chickens. Further research is needed to explore the specific function of this enhancer on *tnni1b*, such as the specific expression site of *tnni1b* driven by this enhancer, and the role of the enhancer in heart development or tissue homeostasis.

### Conclusions

We identified an 87 bp heart-specific enhancer, tnni1b-ECR183-d2, which is located 84 kb upstream of the heart development-related gene *tnni1b*. The enhancer activity is positively correlated with NKX2.5 or JUN. Although the zebrafish and human enhancers in this study target different tissues, this 87 bp zebrafish enhancer is capable of dominantly driving the specific GFP expression near the atrioventricular junction of the heart. Therefore, the fluorescent label of zebrafish lines mediated by this small but functional heart-specific enhancer is expected to be helpful for cardiovascular research. Further studies are needed to explore the specific regulation of tnni1b-ECR183-d2 on *tnni1b*.

##  Supplemental Information

10.7717/peerj.10289/supp-1 Supplemental Information 1 Information of embryo numbers and GFP expression from transient injections of tnni1b-ECR183, tnni1b-ECR183-d1, tnni1b-ECR183-d2, tnni1b-ECR183-d3,tnni1b-ECR183-h179 and tnni1b-ECR183-h84

10.7717/peerj.10289/supp-2 Supplemental Information 2 Putative transcription factors binding sites (TFBSs) of tnni1b-ECR183 in PROMO and JASPAR

10.7717/peerj.10289/supp-3 Supplemental Information 3 Identification of zebrafish enhancer activity and analysis of putative TFBSs by luciferase reporter assay

10.7717/peerj.10289/supp-4 Supplemental Information 4 Identification of human enhancer activity and analysis of putative TFBSs by luciferase reporter assay

10.7717/peerj.10289/supp-5 Figure S1 Lateral views of zebrafish embryos with GFP expression outside of the heart after injection with tol2 mRNA and tnni1b-ECR183 Scale bars = 100 µm.

10.7717/peerj.10289/supp-6 Figure S2 Binding reactions between tnni1b-ECR183-d2 and NKX2.5 (lanes 1–4), ETS1 (lanes 5–8) and JUN (lanes 9–12) based on the EMSA Lanes 13-15 show the binding reactions of the control EBNA system. Specific binding reactions are shown in lanes 1, 5, 9 and 13; competition reactions are shown in lanes 2, 6, 10 and 14; mutation reactions are shown in lanes 3, 7 and 11; and negative reactions are shown in lanes 4, 8, 12 and 15.

10.7717/peerj.10289/supp-7 Table S1 Primers of oligos for target DNA synthesis in EMSA

10.7717/peerj.10289/supp-8 Table S2 Information on 16 ECRs in the 219 kb zebrafish genomic region

10.7717/peerj.10289/supp-9 Video S1 Heart-specific GFP expression of embryos injected with tnni1b-ECR183-d2 at 24 hpf

10.7717/peerj.10289/supp-10 Video S2 Heart-specific GFP expression of embryos injected with tnni1b-ECR183-d2 at 48 hpf

10.7717/peerj.10289/supp-11 Video S3 Heart-specific GFP expression of embryos injected with tnni1b-ECR183-d2 at 72 hpf

## Additional Information and Declarations

### Data Availability

The following information was supplied regarding data availability: The raw data are available in the Supplementary Files.
