# Supplementary figures and images for: Tnni1b-ECR183-d2, an 87 bp cardiac enhancer of zebrafish
Source: PeerJ. 2020 Nov 4;8:e10289. doi: 10.7717/peerj.10289 (PMC7648457; doi:10.7717/peerj.10289)

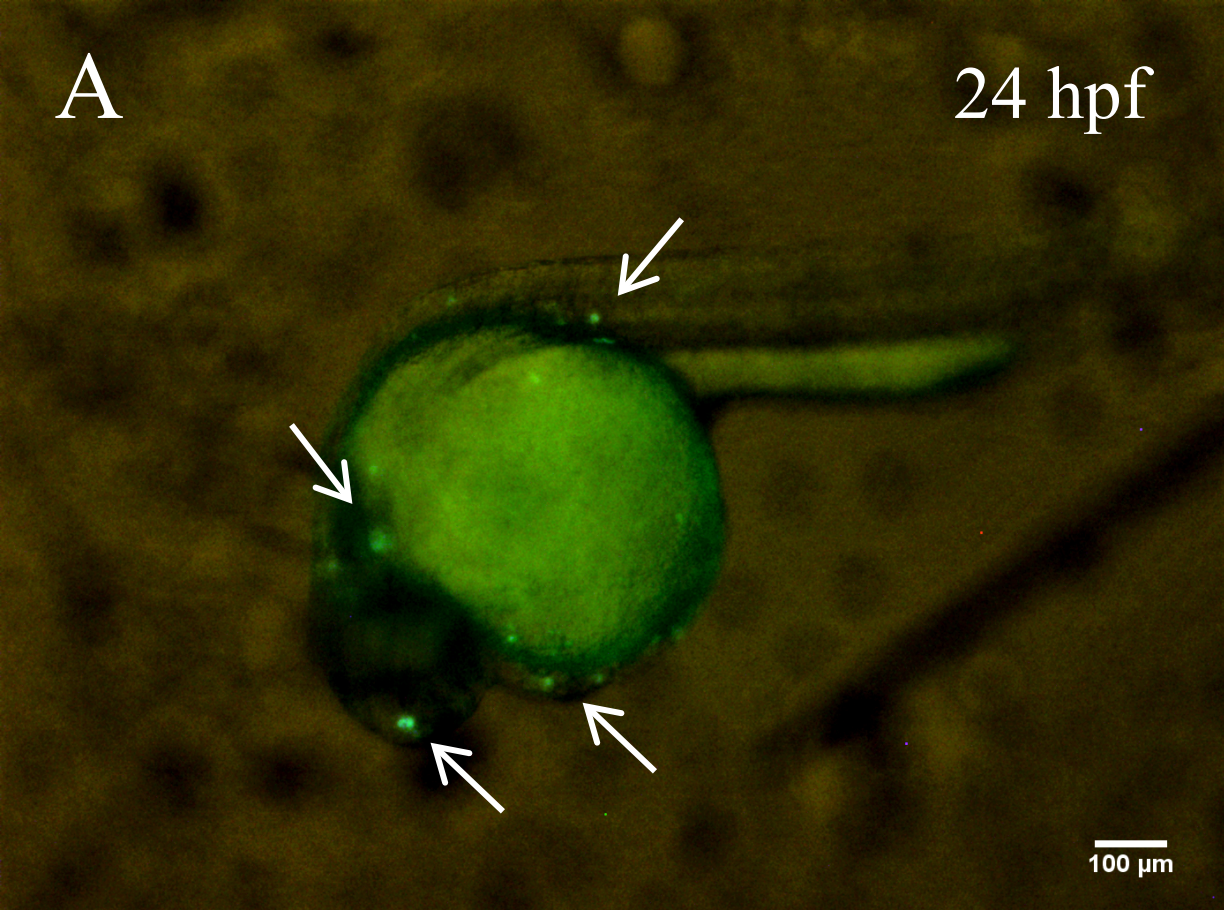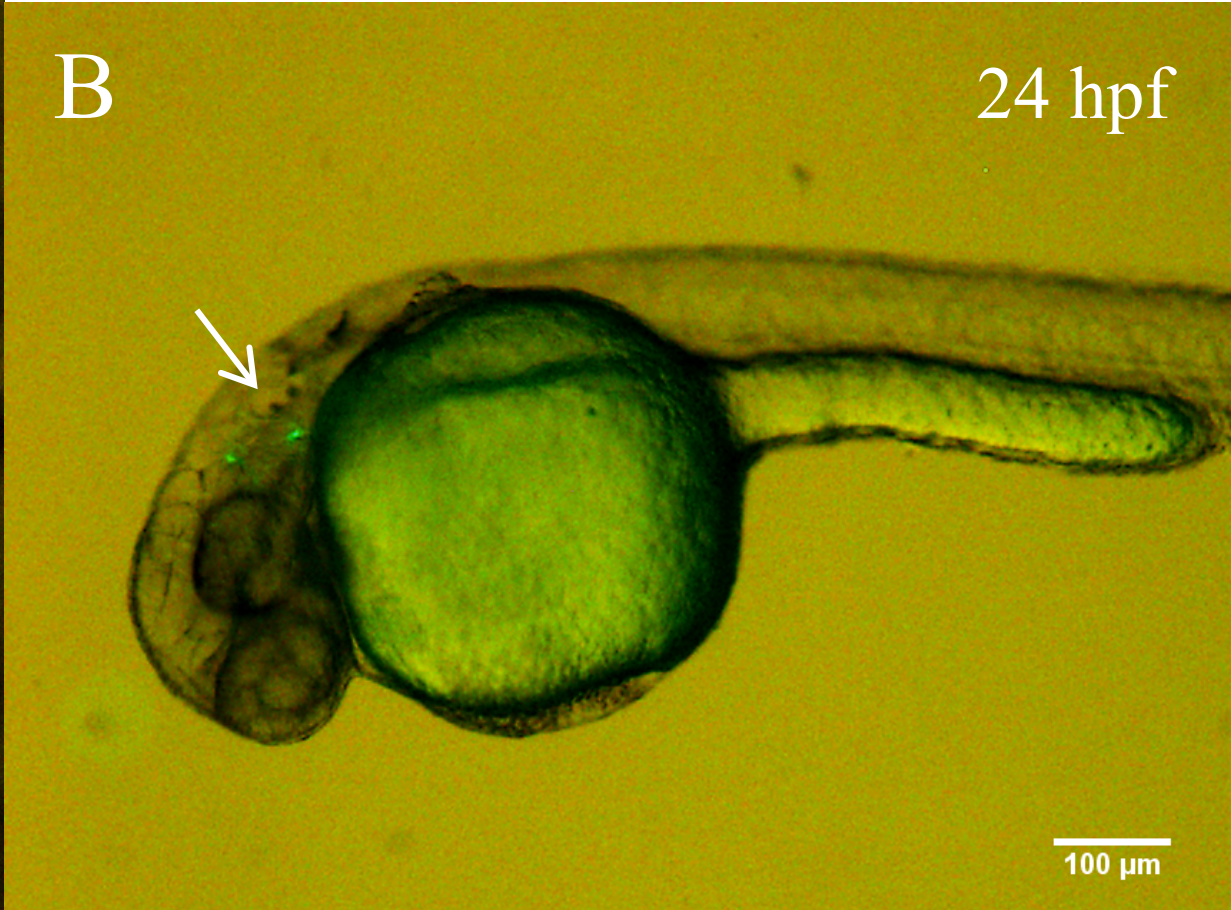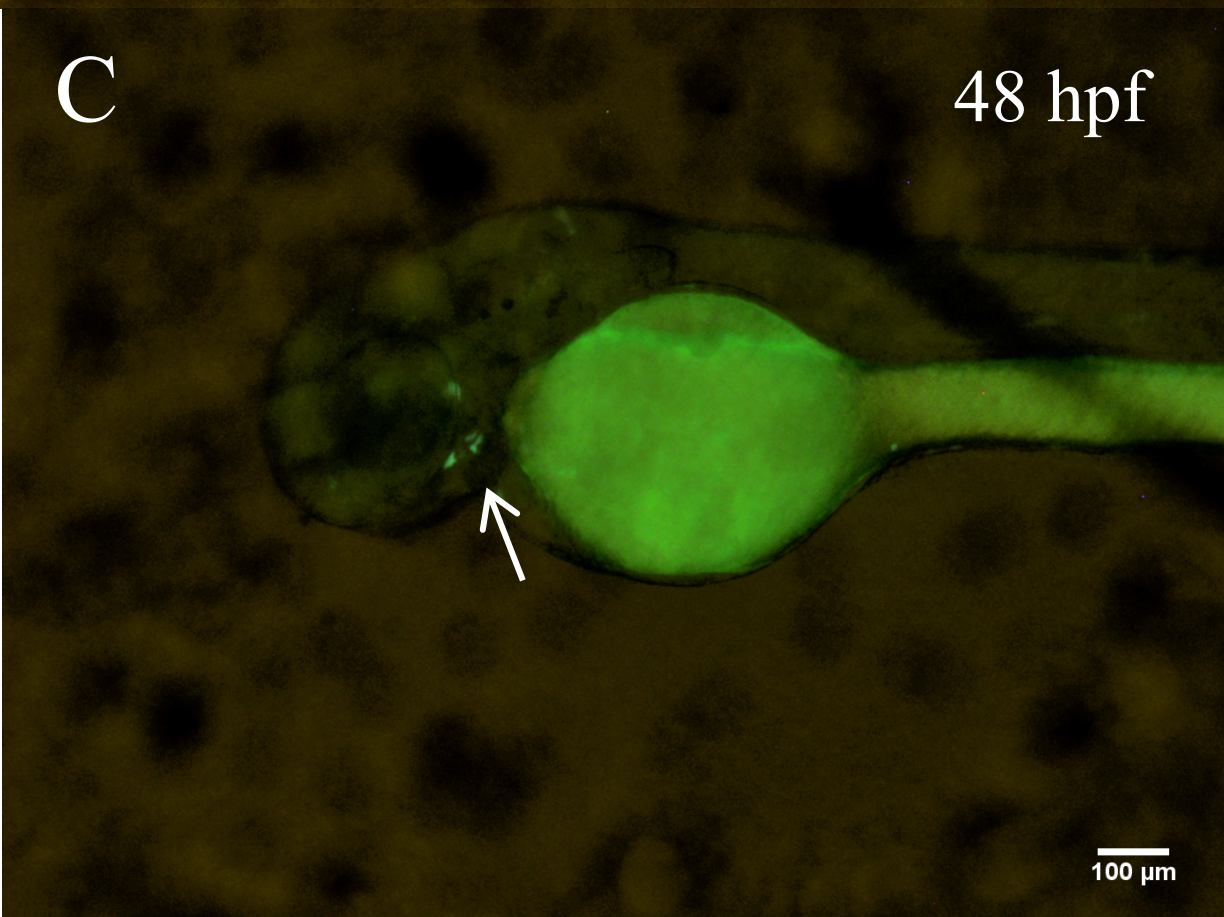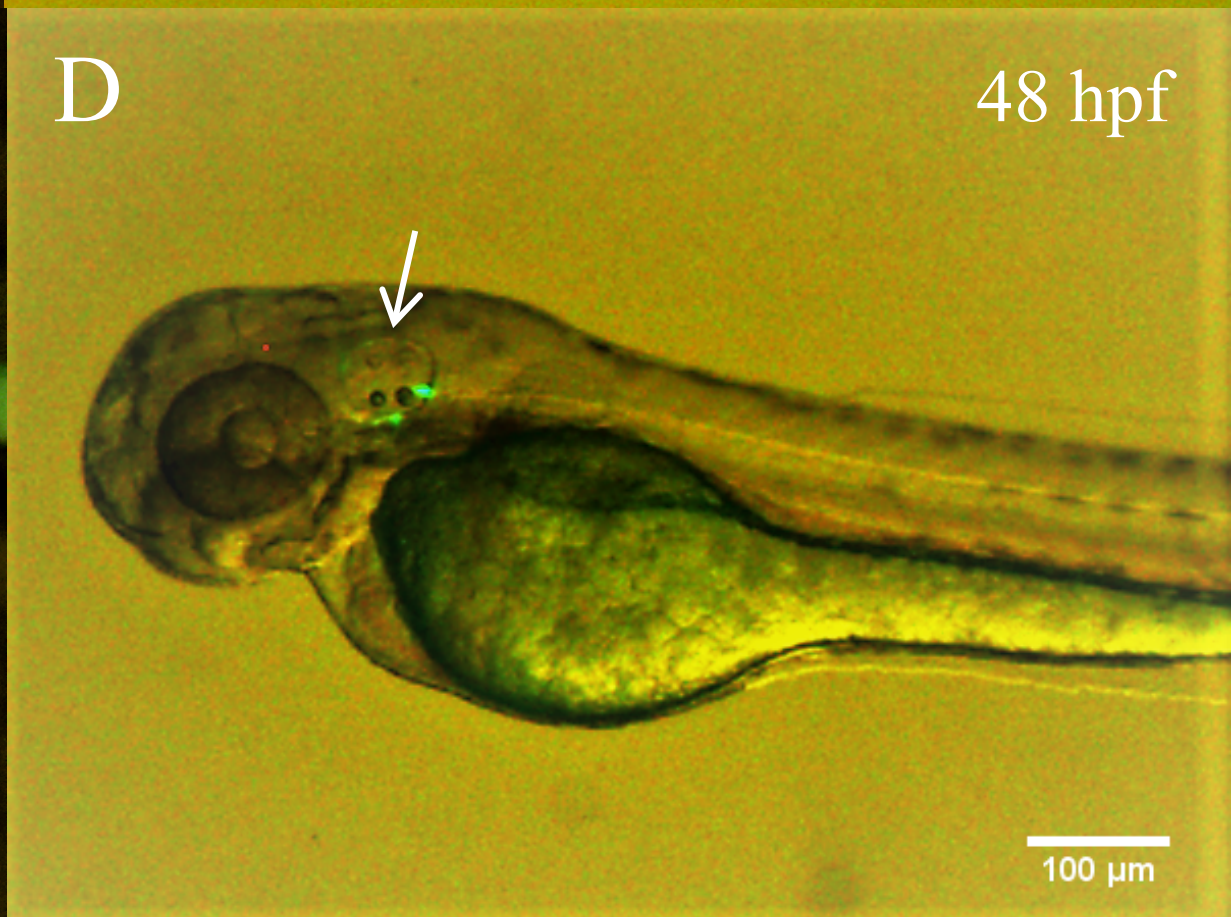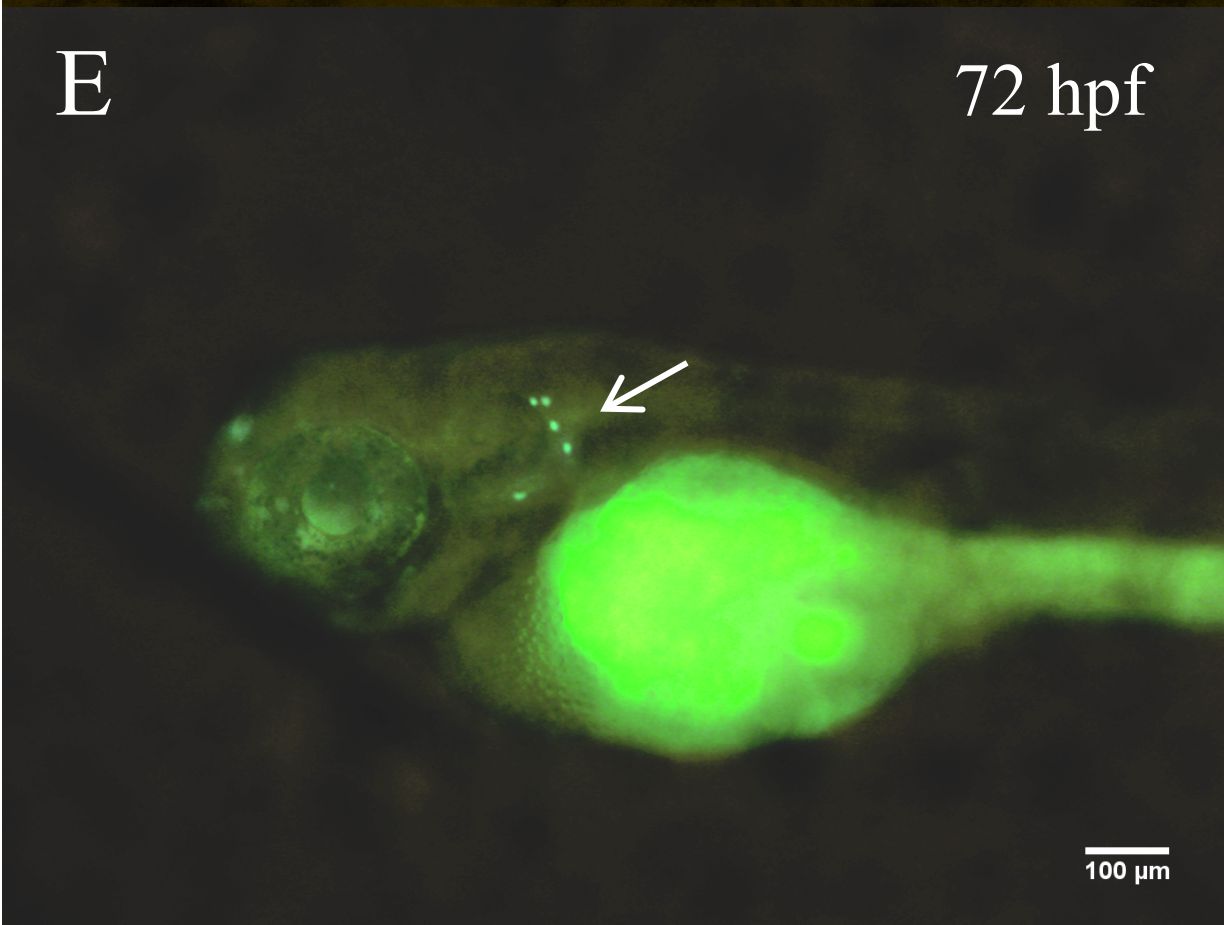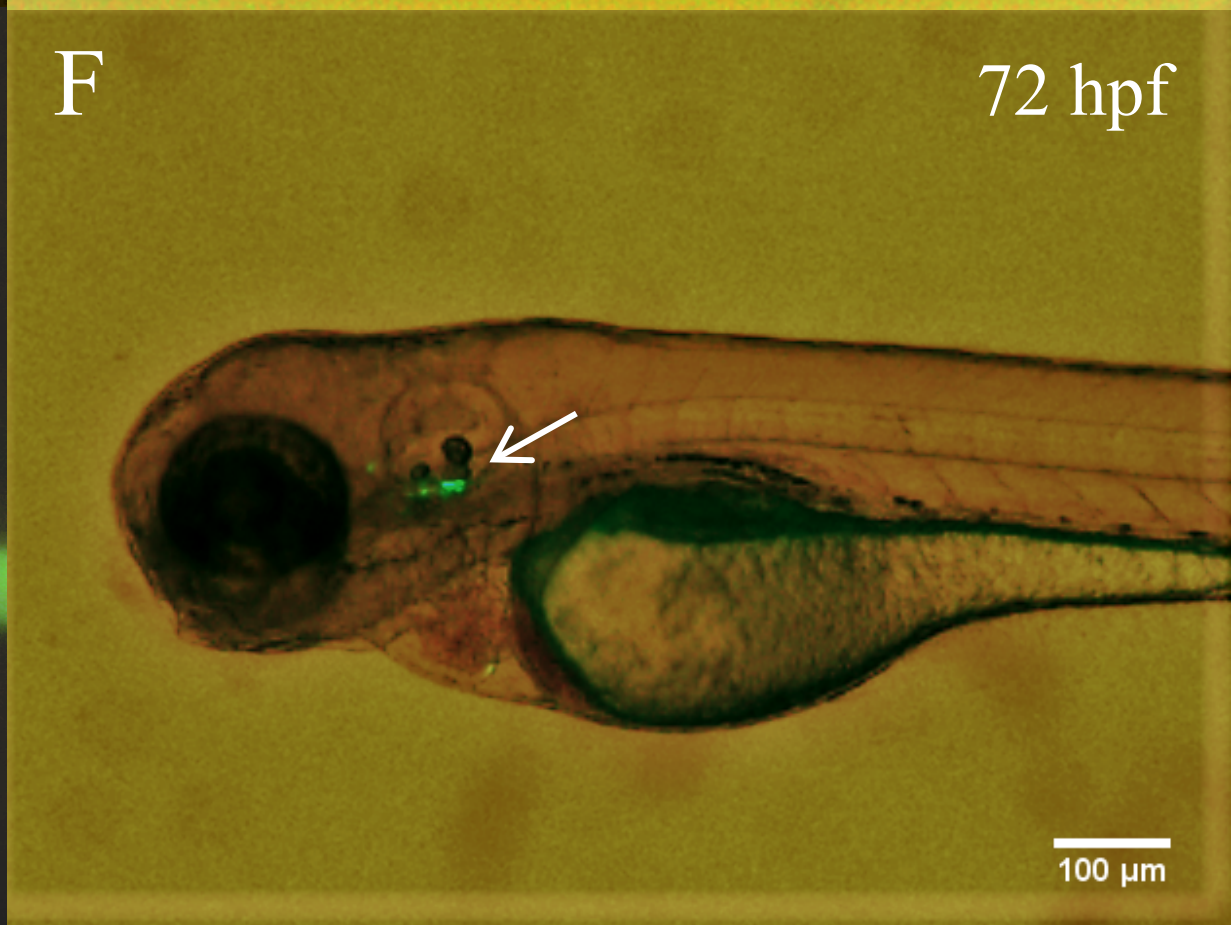

Supplement: Figure S1 — Scale bars = 100 µm. [file peerj-08-10289-s005.pdf]

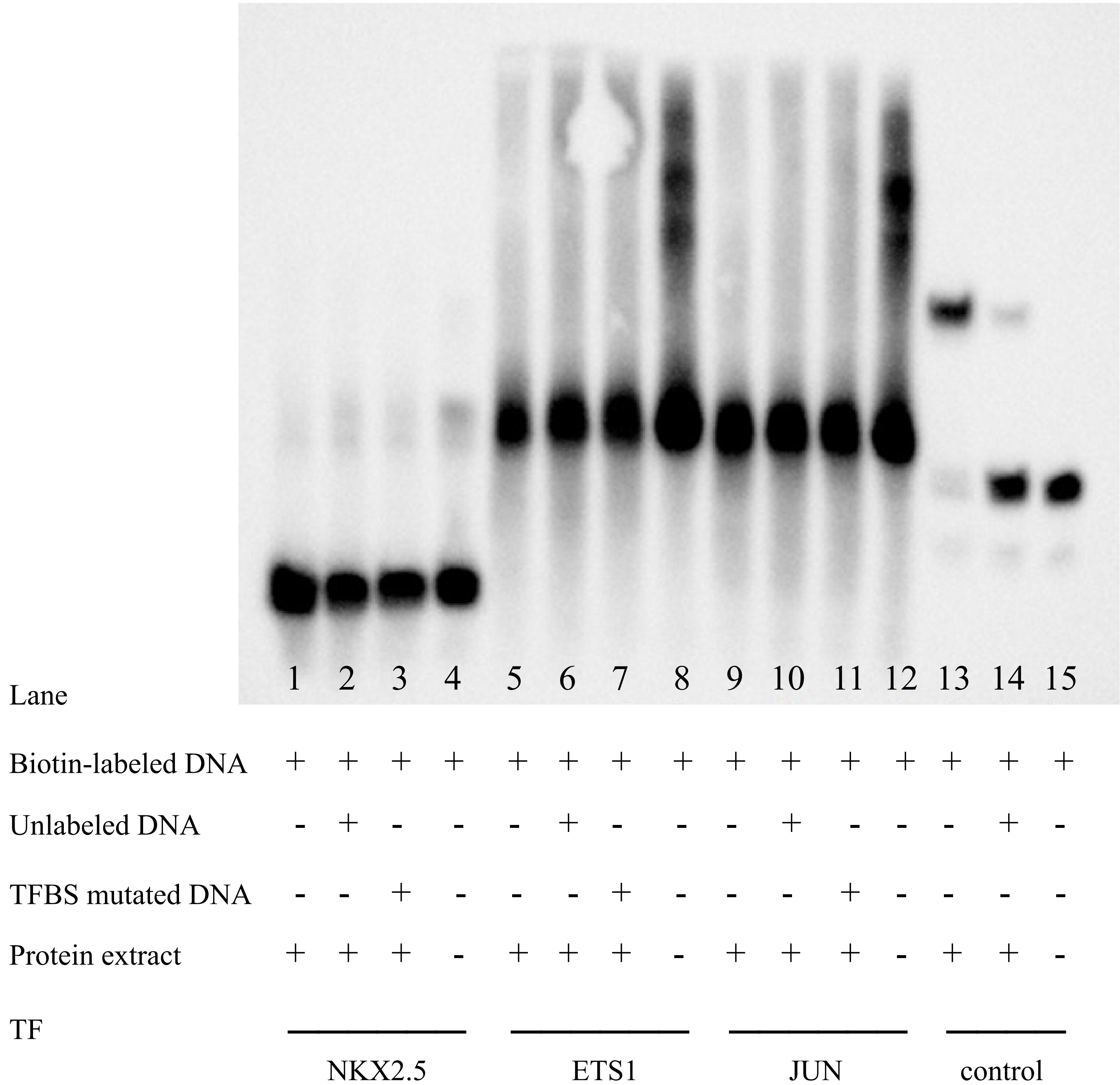

Supplement: Figure S2 — Lanes 13-15 show the binding reactions of the control EBNA system. Specific binding reactions are shown in lanes 1, 5, 9 and 13; competition reactions are shown in lanes 2, 6, 10 and 14; mutation reactions are shown in lanes 3, 7 and 11; and negative reactions are shown in lanes 4, 8, 12 and 15. [file peerj-08-10289-s006.pdf]
