# Supplementary material for: Tnni1b-ECR183-d2, an 87 bp cardiac enhancer of zebrafish
Source: PeerJ. 2020 Nov 4;8:e10289. doi: 10.7717/peerj.10289 (PMC7648457; doi:10.7717/peerj.10289)
Supplement: Table S1 [file peerj-08-10289-s007.docx]

Table S1. Primers of oligos for target DNA synthesis in EMSA

| **Target DNA** | **Sequences (5’-3’)** |
| --- | --- |
| NKX2.5-5’ biotin-labeled | F: tagaggaaggagcatctccggcttggagacg  R: cgtctccaagccggagatgctccttcctcta |
| NKX2.5-  binding site mutated | F: tagaggaaggagcatc**g**(t)ccggcttggagacg  R: cgtctccaagccgg**c**(a)gatgctccttcctcta |
| ETS1-5’  biotin-labeled | F: agctgctgccggtagaggaaggagcatctccggcttggagacgaggactggactgaccgtg  gcgccaggagagaggag  R: ctcctctctcctggcgccacggtcagtccagtcctcgtctccaagccggagatgctccttcctcta  ccggcagcagct |
| ETS1-  binding site mutated | F: agctgctgc**t**(c)ggtagaggaaggagcatc**g**(t)ccggcttggagacgaggactggactgac  cgtggcgcca**a**(g)gagagaggag  R: ctcctctctc**t**(c)tggcgccacggtcagtccagtcctcgtctccaagccgg**c**(a)gatgctccttc  ctctacc**a**(g)gcagcagct |
| JUN-5’  biotin-labeled | F: ctgacagatagctgctgccggtagaggaaggagcatctccggcttggagacgaggactggac  tgaccgtggcgc  R: gcgccacggtcagtccagtcctcgtctccaagccggagatgctccttcctctaccggcagcagc  tatctgtcag |
| JUN-  binding site mutated | F: ctga**t**(c)agatagctgctgccggtagaggaaggagcatctccggcttggagacgaggactg  gac**g**(t)gaccgtggcgc  R: gcgccacggtc**c**(a)gtccagtcctcgtctccaagccggagatgctccttcctctaccggcagc  agctatct**a**(g)tcag |

F：Forward primer sequence

R：Reverse primer sequence

Mutant bases are shown as bolded text, original bases were in parentheses.
