# Supplementary material for: Tnni1b-ECR183-d2, an 87 bp cardiac enhancer of zebrafish
Source: PeerJ. 2020 Nov 4;8:e10289. doi: 10.7717/peerj.10289 (PMC7648457; doi:10.7717/peerj.10289)
Supplement: Table S2 [file peerj-08-10289-s008.docx]

Table S2. Information on 16 ECRs in the 219 kb zebrafish genomic region.

| **No.** | **Location** | **Length (bp)** | **Percent identity (%)** | **Distance from *tnni1b* (kb)** | **Notes** |
| --- | --- | --- | --- | --- | --- |
| 1 | chr6:54703347-54703462 | 116 | 91.4 | 77 | with transposable elements and simple repeats |
| 2 | chr6:54705867-54706081 | 215 | 85.1 | 75 | with transposable elements and simple repeats |
| 3 | chr6:54723557-54723684 | 128 | 74.2 | 57 | with transposable elements and simple repeats |
| 4 | chr6:54747381-54747508 | 128 | 74.2 | 33 | with transposable elements and simple repeats |
| 5 | chr6:54805911-54806020 | 110 | 72.7 | 6 | with transposable elements and simple repeats |
| 6 | chr6:54810111-54810274 | 164 | 80.5 | 10 | with transposable elements and simple repeats |
| 7 | chr6:54820405-54820584 | 180 | 75 | 21 | with transposable elements and simple repeats |
| 8 | chr6:54851640-54851887 | 248 | 76.6 | 52 | with transposable elements and simple repeats |
| 9 | chr6:54856297-54856418 | 122 | 73 | 57 | with transposable elements and simple repeats |
| 10 | chr6:54879204-54879304 | 101 | 70.3 | 79 | with transposable elements and simple repeats |
| 11 | chr6:54883657-54883839 | 183 | 71.6 | 84 | termed tnni1b-ECR183 in text |
| 12 | chr6:54885280-54885470 | 191 | 80.1 | 86 | with transposable elements and simple repeats |
| 13 | chr6:54891814-54892059 | 246 | 76.4 | 92 | with transposable elements and simple repeats |
| 14 | chr6:54703347-54703462 | 116 | 91.4 | 77 | with transposable elements and simple repeats |
| 15 | chr6:54705867-54706081 | 215 | 85.1 | 75 | with transposable elements and simple repeats |
| 16 | chr6:54723557-54723684 | 128 | 74.2 | 57 | with transposable elements and simple repeats |
